# Expanding Primary Metabolism Helps Generate the Metabolic Robustness To Facilitate Antibiotic Biosynthesis in *Streptomyces*

**DOI:** 10.1128/mBio.02283-17

**Published:** 2018-02-06

**Authors:** Jana K. Schniete, Pablo Cruz-Morales, Nelly Selem-Mojica, Lorena T. Fernández-Martínez, Iain S. Hunter, Francisco Barona-Gómez, Paul A. Hoskisson

**Affiliations:** a Strathclyde Institute of Pharmacy and Biomedical Sciences, University of Strathclyde, Glasgow, United Kingdom; b Evolution of Metabolic Diversity Laboratory, Langebio, Guanajuato, Mexico; c Department of Biology, Edge Hill University, Ormskirk, Lancashire, United Kingdom; Korea Advanced Institute of Science and Technology

**Keywords:** actinobacteria, primary metabolism, *Streptomyces*, antibiotics, evolution, pyruvate kinase

## Abstract

The expansion of the genetic repertoire of an organism by gene duplication or horizontal gene transfer (HGT) can aid adaptation. *Streptomyces* bacteria are prolific producers of bioactive specialized metabolites that have adaptive functions in nature and have found extensive utility in human medicine. While the biosynthesis of these specialized metabolites is directed by dedicated biosynthetic gene clusters, little attention has been focused on how these organisms have evolved robustness in their genomes to facilitate the metabolic plasticity required to provide chemical precursors for biosynthesis during the complex metabolic transitions from vegetative growth to specialized metabolite production and sporulation. Here, we examine genetic redundancy in actinobacteria and show that specialized metabolite-producing bacterial families exhibit gene family expansion in primary metabolism. Focusing on a gene duplication event, we show that the two pyruvate kinases in the genome of *Streptomyces coelicolor* arose by an ancient duplication event and that each has evolved altered enzymatic kinetics, with Pyk1 having a 20-fold-higher *k*_cat_ than Pyk2 (4,703 s^−1^ compared to 215 s^−1^, respectively), and yet both are constitutively expressed. The pyruvate kinase mutants were also found to be compromised in terms of fitness compared to wild-type *Streptomyces*. These data suggest that expanding gene families can help maintain cell functionality during metabolic perturbation such as nutrient limitation and/or specialized metabolite production.

## IMPORTANCE

The rise of antimicrobial-resistant infections has prompted a resurgence in interest in understanding the production of specialized metabolites, such as antibiotics, by *Streptomyces*. The presence of multiple genes encoding the same enzymatic function is an aspect of *Streptomyces* biology that has received little attention; however, understanding how the metabolic expansion influences these organisms can help enhance production of clinically useful molecules. Here, we show that expanding the number of pyruvate kinases enables metabolic adaptation, increases strain fitness, and represents an excellent target for metabolic engineering of industrial specialized metabolite-producing bacteria and the activation of cryptic specialized metabolites.

## INTRODUCTION

A remarkable feature of specialized metabolite-producing actinobacterial genomes is the annotation of multiple genes that encode the same putative biochemical function (1, 2). This expansion of gene families by gene duplication or horizontal gene transfer (HGT) is thought to introduce robustness into biological systems, which in turn facilitates evolvability and adaptation (3–5). The expansion of gene families results in relaxed selection following the gene duplication or HGT event, which allows the accumulation of mutations which enable diversification of function to occur (6). This suggests that gene family expansion within genomes is a key driver of biological innovation by facilitating adaptation (7). The production of extensive specialized metabolites by certain actinobacterial lineages is thought to be a key adaptive response to life in complex, highly competitive environments such as soil (8–10) and, as such, may drive the expansion of primary metabolic capability, providing the metabolic robustness that facilitates the evolution of novel biosynthetic functions.

Surprisingly, many central metabolic enzymes are nonessential for survival due to genetic redundancy through the presence of isoenzymes or metabolic bypasses. The redundancy allows cells to adapt to a variety of habitats and dynamic environmental conditions through provision of metabolic plasticity (11). While this has been studied in the unicellular enteric bacterium *Escherichia coli* and the yeast *Saccharomyces cerevisiae* (12), little attention has been paid to organisms with an extensive specialized metabolism such as *Streptomyces*. Gene families of actinobacterial developmental genes have been studied at the genetic level (7, 13, 14), but little attention has been paid to either central or specialized metabolism (15) and how the supply of biosynthetic precursors is maintained during the adaptive response under challenging environmental conditions.

In actinobacteria, production of specialized metabolites is frequently growth phase dependent and usually in response to nutrient starvation and during entry into the sporulation phase of the life cycle (16). This creates a potential metabolic conflict for an organism, where declining availability of metabolites may constrain certain cellular processes in favor of others, such as reducing cellular pools of metabolites that are used directly for specialized metabolite biosynthesis. Under these conditions, it is likely that genetic redundancy can promote robustness and plasticity that helps to maintain cellular function in the face of perturbation (17, 18), while allowing sporulation and specialized metabolite production to occur.

Here, we systematically examine the genetic redundancy within the genomes of specialized metabolite-producing actinobacteria to understand how genetic robustness enables the evolution of extensive specialized metabolism. Moreover, a detailed functional analysis of a redundant, duplicated pyruvate kinase (PK) gene pair from *Streptomyces coelicolor* A3(2) indicates that biochemical diversification at the enzyme level facilitates the evolution of distinct physiological roles which enable functionality during the metabolic reprogramming that is associated with physiological differentiation and increased fitness.

## RESULTS

### Gene expansion events are overrepresented in specialized metabolite-producing actinobacteria.

To determine if gene expansion events in central metabolism occur with greater frequency in specialized metabolite-producing organisms, a database of 612 actinobacterial genomes spanning 80 genera was compiled (see Table S1 at https://doi.org/10.6084/m9.figshare.5772963.v1). All genomes were retrieved from GenBank, reannotated with RAST (19) to ensure consistency of annotation across the database, and then analyzed in a bespoke bioinformatics pipeline based on EvoMining (20, 21) (see Fig. S1 and Table S1 at https://doi.org/10.6084/m9.figshare.5772963.v1). It was hypothesized that, if precursor-supplying pathways are a contributing factor to the adaptive response of specialized metabolite production, then the enzymatic nodes contributing to precursor supply should be overrepresented in the database (Table 1; also see Tables S1 and S2 at https://doi.org/10.6084/m9.figshare.5772963.v1).

**TABLE 1:** Percentage of primary metabolic pathway gene expansion per suborder and pathway of central carbon metabolism^a^

Order/suborder	No. of genera	No. of species	% expansion per pathway (no. of enzymes in minimum pathway):									
			GLY (10)	GNG (5)	PPP (7)	TCA (17)	AA derived from:					
							2OG (13)	PYR (10)	OAA (18)	3PGA (7)	R5P (10)	E4P/PEP (18)
*Streptomycineae*	3	303	**23.3**	8.3	4.8	8.3	11.9	11.1	10.5	16.7	NE	14.0
*Catenulisporineae*	2	3	**25.0**	12.5	**42.9**	25.0	**21.4**	16.7	13.2	**25.0**	10.0	15.8
*Streptosporangineae*	9	28	6.7	11.1	3.2	13.2	10.3	17.3	11.7	11.1	3.3	12.3
*Frankineae*	3	11	6.7	NE	23.8	NE	11.9	NE	8.8	11.1	10.0	8.8
*Pseudonocardineae*	16	57	10.6	**25.0**	17.0	**28.3**	**20.1**	**31.3**	**20.7**	**24.0**	17.5	**19.4**
*Corynebacterineae*	8	62	7.5	9.4	5.4	10.2	8.7	8.3	6.6	6.3	7.5	5.3
*Micromonosporineae*	9	19	20.0	13.9	15.9	16.7	14.3	18.5	**21.1**	13.0	**23.3**	13.5
*Glycomycineae*	1	3	NE	0.0	14.3	NE	7.1	NE	5.3	NE	NE	NE
*Micrococcineae*	17	74	6.5	5.9	5.0	5.5	4.6	2.6	8.4	6.9	3.5	4.0
Bifidobacteriales*	1	3	NE	NE	14.3	NE	0.0	11.1	**21.1**	NE	10.0	NE
*Actinomycineae*	2	26	NE	NE	7.1	NE	0.0	NE	NE	NE	NE	5.3
*Kineosporiineae*	1	1	20.0	NE	28.6	NE	14.3	NE	**21.1**	NE	20.0	5.3
*Propionibacterineae*	8	24	13.8	12.5	7.1	9.4	7.1	18.1	11.8	12.5	7.5	4.6

a The highest percentage of gene expansion for each pathway is highlighted in bold. Abbreviations: GLY, glycolysis; GNG, gluconeogenesis; PPP, pentose phosphate pathway; TCA, tricarboxylic acid cycle; AA, amino acids; 2OG, 2-oxoglutarate; PYR, pyruvate; OAA, oxaloacetate; 3PGA, 3-phosphoglycerate; R5P, ribose-5-phosphate; E4P, erythrose-4-phosphate; PEP phosphoenolpyruvate; NE, no expansion. *, order *Bifidobacteriales*.

Expansion events were defined as cases where the number of enzyme family members per suborder had a value equal to or higher than the mean number of members per phylum plus its standard deviation. The glycolytic pathway showed the highest number of gene expansion events, in the *Streptomycineae* and *Catenulisporineae*, with 23.3% and 25.0% more genes encoding glycolytic function than was average for that pathway in the phylum *Actinobacteria*, respectively (Table 1). *Pseudonocardineae* showed the highest number of gene expansions in gluconeogenesis (25% higher than the mean phylum value) and in the tricarboxylic acid (TCA) cycle (28.3% higher than the mean phylum value). This was also true for many amino acid biosynthetic pathways. Where the main precursor is derived from 2-oxoglutarate (Glu, Gln, Pro, and Arg), expansion was 20.1% more than the mean suborder value, and expansion was 31.3% more with pyruvate-derived amino acids (Ala, Ile, Leu, and Val), 20.7% more with oxaloacetate-derived amino acids (Asp, Asn, Thr, Met, and Lys), 24% more with 3-phosphoglycerate (3PGA)-derived amino acids (Gly, Ser, and Cys), and 19.4% more with erythrose-4-phosphate (E4P)/phosphoenolpyruvate (PEP)-derived amino acids (Tyr, Phe, and Trp).

Focusing on the genus *Streptomyces*, which is renowned as being among the most talented of genera in terms of specialized metabolite production, it was found that 14 enzymatic steps from central metabolism (glycolysis, TCA cycle, and amino acid metabolism) represented gene expansion events, such that they are overrepresented in this genus compared to the rest of the database. The following enzyme functions were found to be overrepresented in the genus *Streptomyces* compared to the whole actinobacterial phylum: phosphofructokinase (PFK), pyruvate kinase (PK), pyruvate phosphate dikinase (PPDK), malic enzyme (ME), pyruvate dehydrogenase complex E1 (PDHC E1), chorismate mutase, acetylglutamate kinase, diaminopimelate decarboxylase, aspartate aminotransferase, aspartate-semialdehyde dehydrogenase, serine hydroxymethyltransferase, glutamine synthetase, argininosuccinate lyase, and methionine synthetase (see Table S2 at https://doi.org/10.6084/m9.figshare.5772963.v1). Phylogenetic analysis of these expansions indicated that only two gene expansion events are the result of duplication: pyruvate kinase and phosphofructokinase (*pfkA2* and *pfkA3*; previously characterized by Borodina et al. [22]; a phylogenetic analysis of *pfk* is provided in Fig. S4 at https://doi.org/10.6084/m9.figshare.5772963.v1). The remaining expansion events in primary metabolism appear to have arisen through HGT. To investigate how gene expansions through duplication in *Actinobacteria* are a potential prerequisite for increasing robustness in specialized metabolism capability, the duplicated pyruvate kinases from *Streptomyces* were studied further due to their central role in carbon metabolism linking glycolysis, gluconeogenesis, and the TCA cycle.

### Pyruvate kinases in *Streptomyces* arose by gene duplication.

**FIG 1:**
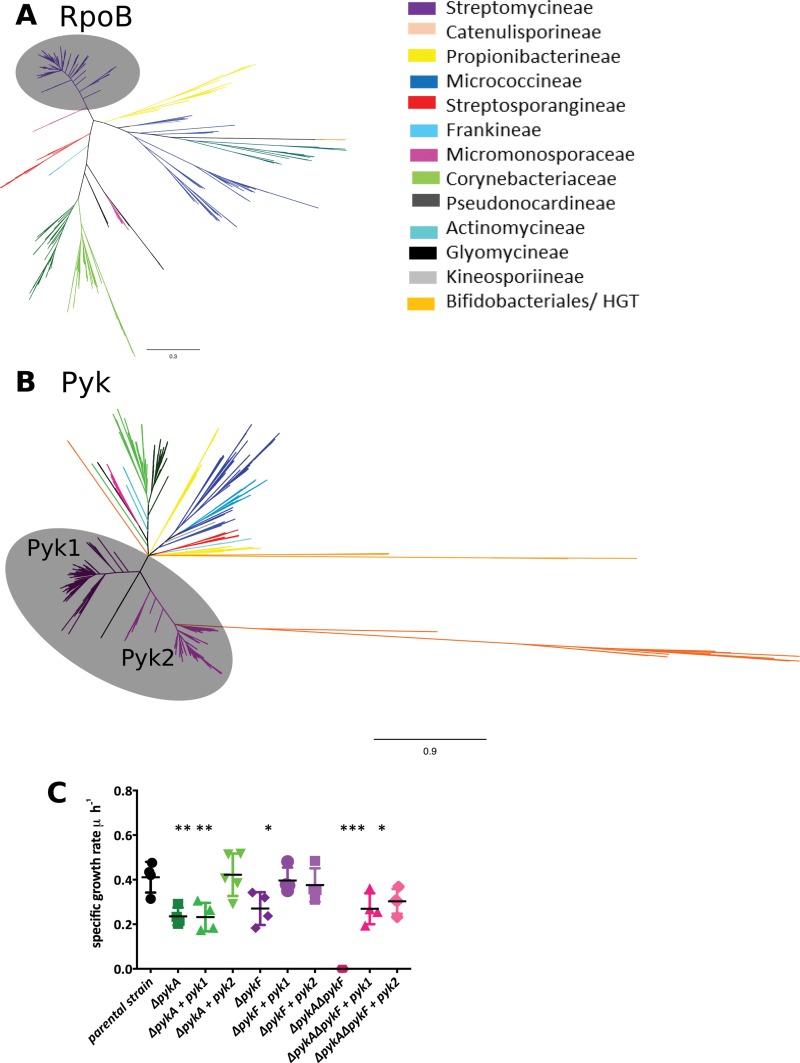
Phylogenetic analysis of RpoB and PK across 80 different actinobacterial genera grouped and color coded by order/suborder. (A) Phylogenetic tree based on RpoB protein sequences. (B) Phylogenetic tree based on pyruvate kinase protein sequences. Gray circles indicate the *Streptomycineae.* (C) Specific growth rate of *E. coli* pyruvate kinase mutants (Δ*pykA*, Δ*pykF*, and Δ*pykA* Δ*pykF*) and the mutants complemented with either *pyk1* or *pyk2* from *S. coelicolor* on M9 medium with glucose as carbon source. *, *P* value ≤ 0.05; **, *P* value ≤ 0.01; ***, *P* value ≤ 0.001.

PK catalyzes the terminal step of glycolysis, converting one molecule of phosphoenolpyruvate to one molecule of pyruvate using ADP as the phosphor acceptor, resulting in the production of ATP. PK therefore plays a key role in linking glycolysis and the citric acid cycle. Moreover, it results in the formation of a direct precursor of acetyl coenzyme A (acetyl-CoA), which feeds directly into polyketide specialized metabolites. A high-resolution species-level phylogeny of the *Actinobacteria* was constructed using the β subunit of RNA polymerase (RpoB [23, 24]), which allowed the segregation of the phylum into three distinct phylogenetic branches: one composed of *Streptomycineae* and *Catenulisporineae*; a second including *Propionibacterineae*, *Actinomycineae*, *Bifidobacteriales*, and *Micrococcineae*; and the third one with *Micromonosporineae*, *Glycomycineae*, *Corynebacterineae*, *Pseudonocardineae*, *Frankineae*, and *Streptosporangineae* (Fig. 1A).

A second phylogeny of the annotated PKs of the *Actinobacteria* was constructed. It indicated that there is a high level of congruence with the RpoB phylogeny, as expected for a central metabolic enzyme (Fig. 1B). However, a bifurcating topology within the *Streptomycetaceae* family was found, which contained the two genes encoding the putative PKs. This topology indicates that a gene duplication event occurred, giving rise to two PKs within this group. Analysis of 286 *Streptomyces* species showed that 281 species have duplicate copies of PK, three species possess a single copy (*Streptomyces somaliensis*, *Streptomyces* sp. strain NRRL F5135, and *Streptomyces scabrisporus*), two species have three copies (*Streptomyces olindensis* and *Streptomyces* sp. strain AcH505), and a single species has four copies (*Streptomyces resistomycificus*). Interestingly, *Streptomyces* sp. AcH505 and *S. resistomycificus* had one copy of *pyk* in each main branch of the PK tree and additional copies were found to be phylogenetically distant (orange branches in Fig. 1B), suggesting that these copies were acquired through horizontal gene transfer (HGT). Overall, 92% (302 of 327) of actinobacterial genomes outside the genus *Streptomyces* encoded a single PK, reinforcing the uniqueness of the duplication in this genus (Fig. 1B).

To determine if the duplicate PKs annotated in the *Streptomyces* genome have pyruvate kinase activity, we used the two PKs from the model streptomycete *S. coelicolor* A3(2) in genetic complementation tests of PK mutants of *Escherichia coli*. *E. coli* also has two PKs: a primary enzyme encoded by *pykF*, which is a type I enzyme, regulated allosterically by fructose-1,6-biphosphate (FBP), and a distinct secondary type II PK (*pykA*), regulated allosterically by AMP (25). In *Streptomyces*, both PKs (Pyk1 and Pyk2) are type II enzymes regulated by AMP. To test for functional complementation, *E. coli* mutants (*ΔpykA*, *ΔpykF*, and a *ΔpykAΔpykF* double mutant [see Table S4 at https://doi.org/10.6084/m9.figshare.5772963.v1]) were tested, along with the isogenic parental strain (*E. coli* BW25113) for their ability to grow under a range of physiological conditions (Fig. 1C). In LB (for which PK is dispensable for growth) and M9 plus acetate as the sole carbon source (where PK is also dispensable for growth), little difference was observed in the specific growth rate (hour^−1^) of the strains. When the strains were grown in M9 plus glucose as the sole carbon source (where PK is essential for growth), the *E. coli ΔpykA ΔpykF* double mutant was unable to grow but could be genetically complemented with either *pyk1* (SCO2014) or *pyk2* (SCO5423) from *S. coelicolor*. The individual *E. coli* Δ*pykA* and Δ*pykF* mutants had reduced specific growth rates (around 50% of the rate of the isogenic parent strain) in M9 plus glucose. Genetic complementation with *pyk1* or *pyk2* from *S. coelicolor* was able to restore growth of an *E. coli ΔpykF* mutant as expected. The *E. coli ΔpykA* mutant could be complemented only with *pyk2* from *S. coelicolor*, suggesting a much more limited physiological role for *pykA* in *E. coli* (Fig. 1C). Remarkably, *pyk2* is the closest BLAST homologue of *pykA* in the *S. coelicolor* genome (43% identity). These data confirm that both *pyk1* and *pyk2* from *S. coelicolor* have retained PK activity following the duplication event but that each has diverged and evolved different physiological roles.

Given that the PKs in *S. coelicolor* have diverged following duplication, we assessed the level of selection imposed on the PKs of *Streptomyces* by calculating the ratio of nonsynonymous changes (*dN*) to synonymous changes (*dS*). Twenty PK sequences from 10 *Streptomyces* genomes were chosen to calculate the *dN*, *dS*, and *dN/dS* values. The *dN/dS* ratio for pairs of *pyk* sequences for each of the genomes yielded *dN/dS* ratios ranging from 0.407 to 0.500, suggesting that PKs in *Streptomyces* are under strong purifying selection (see Table S3 at https://doi.org/10.6084/m9.figshare.5772963.v1). Such high levels of purifying selection indicate that the duplication event in *Streptomyces* is likely to be ancient and is consistent with the PK tree topology (Fig. 1B).

### The two pyruvate kinases in *Streptomyces* have distinct physiological roles.

**FIG 2:**
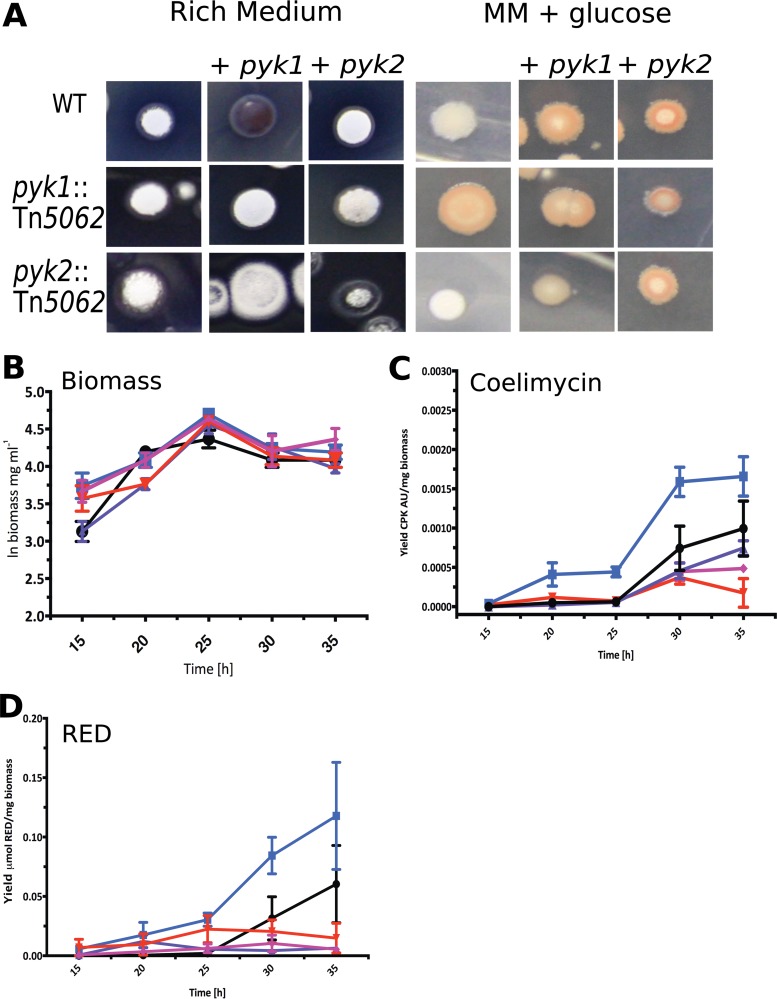
Phenotypic characterization of pyruvate kinase mutants. (A) Wild-type and *pyk1* and *pyk2* transposon mutants of *S. coelicolor* grown on nutrient agar and minimal medium (MM) with glucose, with strains complemented with either *pyk1* or *pyk2* in *trans* and WT strains with additional copies of *pyk1* or *pyk2.* (B) Growth curve of *S. coelicolor* WT, pyruvate kinase mutants, and complemented strains in liquid YEME medium. (C and D) Coelimycin production yield (absorption unit [AU] per milligram of biomass) (C) and undecylprodigiosin (RED) yield during growth in YEME medium (D). Symbols: black circles, WT; blue squares, *pyk1* pyruvate kinase mutant; red inverted triangles, *pyk2* pyruvate kinase mutant; purple triangles, *pyk1*-complemented strain; pink diamonds, *pyk2*-complemented strain.

To determine the roles played by the PKs in growth, development, and antibiotic production, a series of mutant *S. coelicolor* strains was constructed and genetically complemented (see Table S4, Table S5, and Fig. S2 at https://doi.org/10.6084/m9.figshare.5772963.v1). Deletion mutants and transposon insertion mutants showed similar phenotypes (see Fig. S2 at https://doi.org/10.6084/m9.figshare.5772963.v1), and all subsequent work was carried out with transposon insertion mutants. Growth on nutrient agar showed no differences between the strains, except when an additional copy of *pyk1* was present in the wild type (WT) in *trans*, resulting in overproduction of actinorhodin (ACT) in rich medium and undecylprodigiosin (RED) in minimal medium (Fig. 2A). During culture on solid minimal medium with 1% glucose as carbon source, the strains showed no growth defects compared to the wild type (Fig. 2A). Interestingly, the *pyk1*::Tn*5062* mutant showed an increase in specialized metabolite production (Fig. 2A, C, and D). The *pyk2*::Tn*5062* strain was marginally affected in growth and showed no overexpression of specialized metabolites (Fig. 2A). No changes in growth rate were observed in rich medium (YEME medium) for the WT, mutants, or complemented strains (Fig. 2B). However, growth of the strains in this medium showed an increase in production of the polyketide coelimycin (26) and RED in the *pyk1*::Tn*5062* mutant (Fig. 2C), whereas a *pyk2*::Tn*5062* mutant showed reduced antibiotic yields (Fig. 2C and D). These data suggest that each PK isoenzyme plays a distinct physiological role in growth of *Streptomyces* and that perturbation of central metabolism by their deletion or addition affects specialized metabolite biosynthesis. This would suggest that the PKs either are regulated differently at key stages of the *Streptomyces* life cycle at the level of transcription or are regulated at the posttranscriptional/translational level.

**FIG 3:**
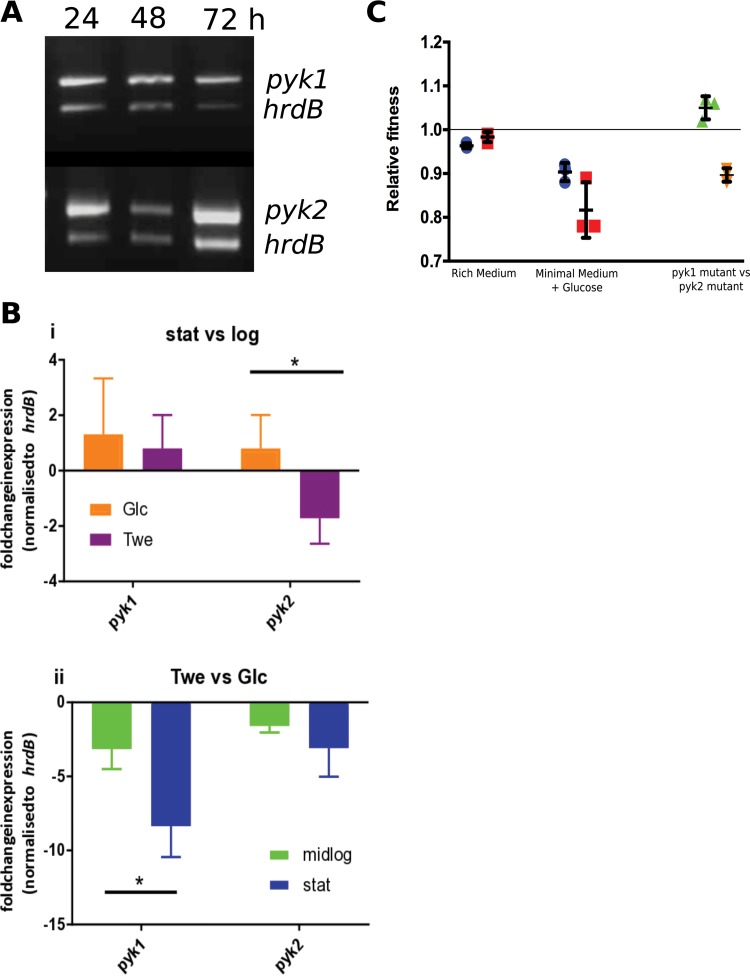
(A) Semiquantitative RT-PCR of expression of *pyk1*, *pyk2*, and *hrdB* throughout the life cycle of *Streptomyces coelicolor*. (B) Fold change expression of *pyk1* and *pyk2* normalized to *hrdB* expression from three biological replicates measuring expression levels by qPCR on growth in minimal medium with either glucose or Tween as carbon source during log or stationary phase comparing expression in stationary phase versus log phase (i) and Tween versus glucose (ii). *, *P* value ≤ 0.05. (C) Fitness of *pyk1* (dark blue circles) and *pyk2* (red squares) mutants compared to WT and each other (relative to *pyk1*) in rich medium (green triangles) and minimal medium with glucose (orange inverted triangles) as sole carbon source. Error bars represent the standard deviation from three biological replicates, with the mean indicated. The line (at a relative fitness of 1.0) indicates the fitness of a strain when competed against itself.

To test this hypothesis, we used semiquantitative reverse transcription-PCR (RT-PCR) to examine the expression of the PKs from *S. coelicolor* throughout growth, relative to the multiplexed vegetative sigma factor encoded by *hrdB*. We found that both genes were constitutively expressed throughout growth (vegetative hyphae and aerial hyphae and during sporulation) relative to *hrdB* (Fig. 3A). To further characterize transcription, we used quantitative real time PCR (qRT-PCR) at two time points during log and stationary phase during either glycolytic growth (glucose as sole carbon source) or anaplerotic/gluconeogenic growth (Tween as sole carbon source [27]) to analyze the expression levels of *pyk1*, *pyk2*, and *hrdB*. Normalizing expression to *hrdB*, there was an expected decrease in *pyk1* expression on Tween compared to glucose during the log and stationary phases of growth (3-fold and 8-fold, respectively [Fig. 3A and B]). Comparison of *pyk1* and *pyk2* during the logarithmic growth phase indicated that *pyk2* had a 1.5-fold-lower level of expression when grown on Tween as the sole carbon source than it did in stationary phase, with all other conditions showing no significant changes in expression between *pyk1* and *pyk2* (Fig. 3B, i), suggesting that activity of PKs in *Streptomyces* is likely to be controlled at the posttranslational level. Expression of *pyk2* also showed a decrease in expression on Tween compared to that on glucose during both phases, but the change was not significant (Fig. 3B, ii).

### Pyk1 and Pyk2 in *Streptomyces* have key substrate affinity differences and specific effector molecules.

To understand the biochemical control of the two PKs of *S. coelicolor*, we purified each enzyme and studied its biochemical characteristics. Both Pyk1 and Pyk2 were activated by the effector molecule AMP, and they both showed Michaelis-Menten-type kinetics for the substrate ADP. For Pyk1, *S*_0.5_ (substrate concentration at half *V*_max_) was 3-fold lower in the presence of 1 mM AMP (0.6 mM down to 0.2 mM [Table 2]), while *V*_max_ also increased 3.5-fold (from 21 U/mg to 73.3 U/mg). Pyk2 showed a 5-fold increase of *V*_max_ in the presence of 1 mM AMP (from 1.2 U/mg to 6.7 U/mg). *S*_0.5_ decreased 3-fold (from 0.3 mM to 0.1 mM [Table 2] [see also Fig. S2 at https://doi.org/10.6084/m9.figshare.5772963.v1]). There were profound differences in the PEP kinetics for the two PKs, with both isoenzymes demonstrating Hill-type cooperative binding kinetics with AMP. In the presence of 1 mM AMP, *V*_max_ of Pyk1 increased 5-fold (14.1 U/mg to 65.45 U/mg), *S*_0.5_ decreased more than 3-fold (3.5 to 1.1 mM), and the Hill coefficient was approximately halved (from 3.7 to 1.8). For Pyk2 in the presence of 1 mM AMP, *V*_max_ was 9.1 U/mg compared to 0.5 U/mg without AMP, with *S*_0.5_ increasing from 1.3 mM to 8.6 mM. Under these conditions, the Hill coefficient increased from 1.5 to 7.1 (Table 2). Further analysis demonstrated that Pyk1 has a much higher affinity for AMP (*S*_0.5_ = 0.01 mM) than does Pyk2 (*S*_0.5_ = 3.8 mM), with a concomitant increase in *V*_max_ (8.2 U/mg for Pyk1 compared to 1 U/mg for Pyk2 [see Fig. S3C at https://doi.org/10.6084/m9.figshare.5772963.v1]). The turnover rate constant (*k*_cat_) for Pyk1 was >20-fold greater (4,703 s^−1^) than that for Pyk2 (215 s^−1^ [Table 2]). Interestingly, Pyk1 was also shown to be highly stimulated by ribose-5-phosphate (see Fig. S3 at https://doi.org/10.6084/m9.figshare.5772963.v1). Intriguingly it is known that flux through the pentose phosphate pathway (PPP) increases during entry into stationary phase in streptomycetes (28), suggesting that Pyk1 activity is stimulated during periods of starvation and during antibiotic production to rebalance reduced glycolytic flux and entry of substrates into the TCA cycle.

**TABLE 2:** Kinetic characteristics of Pyk1 and Pyk2 for the substrates ADP and PEP and the activator AMP

Substrate or activator	Parameter	No AMP		1 mM AMP	
		Pyk1	Pyk2	Pyk1	Pyk2
5 mM PEP with ADP	*V*_max_ (U/mg)	21.0	1.2	73.3	6.7
	*S*_0.5_ (mM)	0.6	0.3	0.2	0.1
	*k*_cat_ (s^−1^)	941	39	4,703	215
	*k*_cat_/*K*_*m*_ (mM^−1^ s^−1^)	1,594	145	31,359	2,388
					
1.5 mM ADP with PEP	*V*_max_ (U/mg)	14.1	0.5	65.5	9.1
	*S*_0.5_ (mM)	3.5	1.3	1.1	8.6
	Hill coefficient	3.7	1.5	1.8	7.1
	*k*_cat_ (s^−1^)	350	18	4,200	336
	*k*_cat_/*S*_0.5_ (mM^−1^ s^−1^)	100	14	4,000	39
					
AMP	*V*_max_ (U/mg)	8.2	1		
	*S*_0.5_ (mM)	0.01	3.8		
	*k*_cat_ (s^−1^)	423.7	42.0		
	*k*_cat_/*S*_0.5_ (mM^−1^ s^−1^)	42,373	11		

### Pyruvate kinase mutants are less fit than wild-type *S. coelicolor*.

Given that the PK gene duplication has been retained in streptomycetes, it would suggest that there is a fitness advantage conferred by having more than one copy of PK. To test this hypothesis, we competed marked WT (with integrated empty pIJ6902, thiostrepton resistant) with *pyk1*::Tn*5062* (apramycin-resistant) and *pyk2*::Tn*5066* (hygromycin-resistant) strains to assess the relative fitness of each strain. In rich medium, where PK should be dispensable for growth, there is no significant difference between WT and PK mutants in terms of fitness (Fig. 3C). When the fitness of the PK mutants was assessed in minimal medium with glucose as the sole carbon source, where PK should be required for growth, strains with a single PK have significantly reduced fitness compared to WT (Fig. 3C) (*pyk1*::Tn*5062*, *P* < 0.05; *pyk2*::Tn*5066*, *P* < 0.01). The distinct physiological roles of Pyk1 and Pyk2 in *Streptomyces* suggest that the relative fitness of each mutant would differ when competed against each other. In rich medium, the *pyk1*::Tn*5062* strain exhibits increased relative fitness compared to the *pyk2*::Tn*5066* strain (Fig. 3C) (*P* < 0.05); however, in minimal medium with glucose as the sole carbon source, the *pyk1*::Tn*5062* strain is less fit than the *pyk2*::Tn*5066* strain (Fig. 3C) (*P* < 0.01). These data demonstrate that possession of two copies of PK confers increased relative fitness to strains of *Streptomyces* under different physiological conditions.

## DISCUSSION

Metabolic robustness is a key biological adaptation to coping with environmental perturbation that enables an organism to persist in an environment (17). The ability of an organism to adapt to dynamic and competitive environments requires minimization of the effects of metabolic perturbations, which can be achieved through gene family expansion following either gene duplication or HGT. During nutrient stress, intracellular concentrations of key metabolic intermediates can fall, which, when coupled with increasing demand during specialized metabolite biosynthesis, may produce metabolic conflict in cells. PK occupies a key position in carbon metabolism, bridging glycolysis to the TCA cycle, and plays a central role in the generation of ATP and precursors for the synthesis of specialized metabolite precursors (acetyl-CoA, amino acids, organic acids, etc. [27]). At key branch points in metabolism, such as here, the levels of metabolites are tightly controlled (29), and in *E. coli*, the flux between PEP and pyruvate is stably maintained by two phylogenetically distinct PKs (25, 30). In the streptomycetes, a duplication event has led to the evolution of altered substrate affinity and enzyme efficiency in the two duplicate copies which contribute to metabolic robustness enabling cellular processes to occur during times of perturbation, such as at the entry into stationary phase and specialized metabolite production.

Enzyme family expansion in central metabolism is widespread in the *Actinobacteria*, in particular with those genera that are extensive producers of specialized metabolites (21) (see Table S1 at https://doi.org/10.6084/m9.figshare.5772963.v1), with the *Streptomycineae* showing extensive enzyme family expansion in glycolysis. Within glycolysis, only two enzyme families show duplication as a route to expansion. Borodina et al. (22) showed that PfkA2 was the primary phosphofructokinase in *S. coelicolor* but did not study the wider role of duplication or a detailed kinetic comparison of the expansion event. The duplication of pyruvate kinase is ancient and has permitted subsequent divergence of the gene pair to evolve distinct physiological roles, where Pyk2 appears to function as a housekeeping PK, with a higher affinity for PEP (when AMP is low), and Pyk1 exhibits strong activation as AMP concentrations rise. An increased AMP concentration is a well-established starvation signal in bacteria (31) and may serve to increase flux through the terminal end of glycolysis during starvation to facilitate precursor supply for specialized metabolites. Moreover, the PPP intermediate ribose-5-phosphate stimulates Pyk1 activity, providing a physiological link between the PPP and the associated generation of NADPH, which has established links to synthesis of specialized metabolites, including those overproduced by strains engineered in this work (28). Disruption of *pyk1* in *S. coelicolor* leads to increased levels of coelimycin and undecylprodigiosin when grown in rich medium (such as in industrial situations) but no significant increase in the yield of the polyketide antibiotic actinorhodin, which may reflect the ranges of different metabolic control points affecting biosynthesis of chemically similar specialized metabolites. The regulation of this node is known to be controlled by at least one global regulator, ScbR2, which is encoded in the coelimycin (*cpk*) biosynthetic gene cluster (BGC). ScbR2 expressed in mid-log phase has been shown to bind to *pyk2* and reduce its expression. ScbR2 is also a regulator of ACT and RED biosynthesis in addition to the angucyclines (32). Interestingly, coelimycin was originally considered to be a cryptic BGC and the disruption of PK activity results in increased production of this molecule, suggesting that strategies that affect precursor supply may activate the plethora of cryptic biosynthetic pathways in *Actinobacteria* and may provide a useful strategy for studying these molecules.

Our data demonstrate that the duplication of pyruvate kinase promotes metabolic robustness through altered kinetic parameters which increase the relative fitness of strains and influence the production of specialized metabolites. Understanding the evolution of central metabolism in conjunction with specialized metabolism can contribute to our fundamental understanding of the ability of *Actinobacteria* to produce a plethora of useful molecules and can help inform on novel approaches to metabolic engineering and genome mining (21).

## MATERIALS AND METHODS

### Database generation and bioinformatics analysis.

The NCBI database (https://www.ncbi.nlm.nih.gov/genbank/wgs) was the source of actinobacterial genomes having a minimum coverage of 25× and less than 30 contigs per mega-base pair. To ensure a wide range of phylogeny, a selection of 612 species from 80 genera were included. Each genome was reannotated using RAST (19), and the annotation files were used to determine the frequency of each functional annotation. The mean number of occurrences of each functional role was calculated per genus, and examples which had a value equal to or higher than the mean plus its standard deviation were defined as a “gene expansion event.” Each candidate protein sequence was extracted from the actinobacterial genome database to form a BLASTP analysis working database. The sequences were then aligned with MUSCLE v3.8.31 (33), and alignments were scrutinized using Jalview v2.10.1 to ensure that at least 25% coverage with the query was achieved; if not, sequences and expansions were discarded from the working database. Phylogenetic analysis of the alignments was conducted using MrBayes (34) v3.2.3 with trees visualized in FigTree v1.4.2 (http://tree.bio.ed.ac.uk/software/figtree/). Sequences were obtained from the NCBI gene database and aligned with ClustalW algorithm in MEGA v6.06 (35), and the synonymous (*dS*) and nonsynonymous (*dN*) changes were determined using the distance model function with the Nei-Gojobori method (36) with the Jukes-Cantor algorithm (37) bootstrap.

### Growth and mutant construction in *Streptomyces*.

A full list of strains, plasmids and oligonucleotides used in this study is provided in Tables S4, S5, and S6 at https://doi.org/10.6084/m9.figshare.5772963.v1. Routine growth, spore generation, and conjugation of *Streptomyces* strains were carried out according to the work of Kieser et al. (38). Antibiotic titers were determined according to the work of Gomez-Escribano et al. (26) for coelimycin and the work of Kieser et al. (38) for RED and ACT. *S. coelicolor* gene knockout mutants (*Δpyk1* and *Δpyk2*) were constructed using PCR-targeted gene replacement with an apramycin resistance cassette [*acc(3)IV*] using the Redirect system (39) and the primers reported in Table S6 at https://doi.org/10.6084/m9.figshare.5772963.v1. *S. coelicolor* transposon insertion mutants (*pyk1*::Tn*5062* and *pyk2*::Tn*5062*) were constructed using Tn*5062* mutagenized cosmids as described in the work of Fernández-Martínez et al. (40). Each cosmid was first verified by restriction analysis before being conjugated into *Streptomyces*. All strains were verified by PCR and sequencing of the respective products. Growth curves were performed at a 400-ml scale in minimal medium (28) with either 1% glucose or Tween 80 as the carbon source in 2-liter flasks containing a metal spring at 30°C and 250 rpm.

### Interspecies complementation.

*E. coli* single mutants were from the Keio collection of *E. coli* BW25113 (41), and the double mutant was constructed using λ Red recombination of *pykF* according to the work of Datsenko and Wanner (42). Complementation studies used the *Streptomyces pyk1* and *pyk2* genes cloned into pET100_TOPO (Invitrogen).

*E. coli* growth curves were carried out in 250-ml flasks with a working volume of 50 ml of either LB or M9 medium with 1% (wt/vol) glucose or 0.4% (wt/vol) sodium acetate as carbon source. Flasks were inoculated from an overnight culture (1% [vol/vol]) including the appropriate antibiotics and 1 mM isopropyl-β-d-thiogalactopyranoside (IPTG) to induce expression of the PKs. Growth was followed as optical density at 600 nm (OD_600_) at 37°C with shaking at 250 rpm. The specific growth rate was determined from the semilogarithmic plot of biomass concentration.

### Protein overexpression and purification.

The coding sequence of *pyk1* was codon optimized for *E. coli* and amplified from the vector pEX-K4 using the primers in Table S6 at https://doi.org/10.6084/m9.figshare.5772963.v1. The native version of *pyk2* was used to amplify the coding sequence using the primers in Table S6 at https://doi.org/10.6084/m9.figshare.5772963.v1. Both coding sequences were cloned into the pET100_TOPO vector (Invitrogen) according to the manufacturer’s instructions. Overexpression of *pyk1* was in *E. coli* Origami B on LB with 1% (wt/vol) glucose at 30°C until an OD_600_ of 0.4 was reached, and the expression was induced with 0.05 mM IPTG at 18°C overnight. Pyk2 overexpression was in *E. coli* Rosetta using autoinduction medium [components per liter: 10 g tryptone, 5 g yeast extract, 3.3 g (NH_4_)_2_SO_4_, 6.8 g KH_2_PO_4_, 7.1 g Na_2_HPO_4_, 0.5 g glucose, 2.0 g α-lactose, 0.15 g MgSO_4_]; cells were grown at 37°C for 2 h and then reduced to 18°C for overnight cultivation. Cells were disrupted by sonication. Pyk1 and Pyk2 were purified by nickel affinity chromatography using HisTrap FF crude (GE Healthcare) with binding buffer (100 mM KH_2_PO_4_ pH 7.2, 10% [vol/vol] glycerol, 100 mM NaCl, 20 mM imidazole). Tagged proteins were eluted with increasing imidazole concentrations (elution buffer: 100 mM KH_2_PO_4_ [pH 7.2], 10% [vol/vol] glycerol, 100 mM NaCl, 1 M imidazole). Fractions (1 ml) were collected, and the highest concentrations of protein were pooled.

Kinetic characteristics of each pyruvate kinase were determined using purified protein samples according to the method of Bergmeyer et al. (43). Assays to determine the enzyme kinetics for each PK under each condition were carried out in triplicate, and results were analyzed using GraphPad Prism using the Michaelis-Menten or Hill equation where appropriate.

### RNA extraction, semiquantitative RT-PCR, and qPCR analysis.

Biomass of *S. coelicolor* came from liquid cultures, and semiquantitative RT-PCR was carried out according to the method of Clark and Hoskisson (7). Total RNA was used as the template for cDNA synthesis using a qPCRBio cDNA synthesis kit (PCR Biosystems). All cDNA samples were diluted to a concentration of 10 ng/µl with each quantitative PCR (qPCR) mixture containing 10 ng of cDNA (1 µl) and were then mixed with 10 µl MasterMix (2× qPCRBio SyGreen mix Lo-ROX kit from PCR Biosystems) and 2.5 µl of each primer to a final reaction volume of 20 µl using the Corbett Research 6000 analyzer (Qiagen).

### Fitness experiments.

Competitive fitness of PK mutants versus WT was estimated based on the work of Lenski et al. (44). Briefly, spores of each strain were separately pregerminated in 2× YT medium (38) for 7 h at 30°C and washed twice in the culture medium to be used in the fitness experiment. Germling OD_600_ was determined, and a 10-ml culture was inoculated with approximately 1 × 10^5^ CFU of each strain. Initial and final strain densities (after 68 h of growth) were determined by plating on nutrient agar with the appropriate selection. The net growth rate of each competitor was calculated from the data, and the relative fitness of a strain is expressed as the ratio of its growth rate to that of its competitor. Assays were performed in triplicate.
